# New Iron Metabolic Pathways and Chelation Targeting Strategies Affecting the Treatment of All Types and Stages of Cancer

**DOI:** 10.3390/ijms232213990

**Published:** 2022-11-13

**Authors:** George J. Kontoghiorghes

**Affiliations:** Postgraduate Research Institute of Science, Technology, Environment and Medicine, 3 Ammochostou Street, Limassol 3021, Cyprus; kontoghiorghes.g.j@pri.ac.cy; Tel.: +357-2627-2076; Fax: +357-2627-2076

**Keywords:** cancer, iron, chelators, chelator–metal complexes, deferiprone, drug design, drug targeting, multi-target drugs, drug combinations, free radicals, ferroptosis

## Abstract

There is new and increasing evidence from in vitro, in vivo and clinical studies implicating the pivotal role of iron and associated metabolic pathways in the initiation, progression and development of cancer and in cancer metastasis. New metabolic and toxicity mechanisms and pathways, as well as genomic, transcription and other factors, have been linked to cancer and many are related to iron. Accordingly, a number of new targets for iron chelators have been identified and characterized in new anticancer strategies, in addition to the classical restriction of/reduction in iron supply, the inhibition of transferrin iron delivery, the inhibition of ribonucleotide reductase in DNA synthesis and high antioxidant potential. The new targets include the removal of excess iron from iron-laden macrophages, which affects anticancer activity; the modulation of ferroptosis; ferritin iron removal and the control of hyperferritinemia; the inhibition of hypoxia related to the role of hypoxia-inducible factor (HIF); modulation of the function of new molecular species such as STEAP4 metalloreductase and the metastasis suppressor N-MYC downstream-regulated gene-1 (NDRG1); modulation of the metabolic pathways of oxidative stress damage affecting mitochondrial function, etc. Many of these new, but also previously known associated iron metabolic pathways appear to affect all stages of cancer, as well as metastasis and drug resistance. Iron-chelating drugs and especially deferiprone (L1), has been shown in many recent studies to fulfill the role of multi-target anticancer drug linked to the above and also other iron targets, and has been proposed for phase II trials in cancer patients. In contrast, lipophilic chelators and their iron complexes are proposed for the induction of ferroptosis in some refractory or recurring tumors in drug resistance and metastasis where effective treatments are absent. There is a need to readdress cancer therapy and include therapeutic strategies targeting multifactorial processes, including the application of multi-targeting drugs involving iron chelators and iron–chelator complexes. New therapeutic protocols including drug combinations with L1 and other chelating drugs could increase anticancer activity, decrease drug resistance and metastasis, improve treatments, reduce toxicity and increase overall survival in cancer patients.

## 1. Introduction

The high rate of morbidity and mortality in many types of cancer classifies the disease in the top group of chronic conditions with fatal outcomes affecting humanity, with about 20 million new cases and 10 million deaths each year in the last few years [1,2,3]. The design of new strategies for the prevention and treatment of cancer is a major challenge for many health and research institutions and investigators worldwide. Many experimental anticancer drugs receive emergency approval or are classified in the orphan drug category because of the low rate of success with the current available therapies in many types and stages of cancer [4].

In general, anticancer therapeutic strategies involve different protocols of surgery, chemotherapy, radiotherapy and their combinations, with the major aim to reduce cancer cell proliferation and cancer growth and metastasis, as well as potentially altogether eliminate the presence of cancer cells in the body. However, this process is very difficult, especially at the later stages of cancer and mostly at the stage of metastasis, which is the leading cause of cancer-related deaths worldwide [5,6]. Metastasis develops following a multistep process that begins initially at the primary tumor site; then, cancer cells travel and migrate to selected distant organs where they form secondary tumors [5,6]. Many molecular, biochemical, environmental and other factors can trigger cancer initiation, progression and metastasis; this includes iron and associated metabolic, genomic, transcriptional, redoxomic and other pathways, all of which appear to play a major role in high cancer mortality [7,8,9,10].

There are different classifications for cancer chemotherapeutics such as those based on the mechanism of action, e.g., antimetabolites (ribonucleotide reductase inhibitors, purine analogs and antagonists, pyrimidine antagonists, oxazsaphosphorines and antifolates), alkylating agents (platinum-based agents, hydrazines and nitrogen mustards), mitotic spindle inhibitors (vinca alkaloids and taxanes), topoisomerase I and II inhibitors, tyrosine kinase inhibitors, proteasome inhibitors, enzymes and antibiotics. In relation to chemotherapy and drug strategies, specific drug protocols are designed whereby anticancer drugs are directed toward various targets in cancer cells or associated pathways, such as those related to key metabolic protein pathways, DNA synthesis, signal transduction, genomic factors, etc. [8,10]. Different anticancer therapeutic strategies are usually designed for the various types and stages of cancer, metastasis and drug resistance. New, biological, immunological and hybrid drugs such as theranostic formulations, metal complexes, liposomal and nanoparticle formulations, mRNA vaccines, immune checkpoint inhibitors, etc., have been introduced in recent years or are being developed, which, in addition to established anticancer drugs and prevention programs, have decreased the rate of mortality only in some types of cancer. However, drug resistance leading to reduced anticancer drug efficacy is becoming a major problem for old and new categories of drugs, affecting 90% of cancer patients [11,12,13,14]. One or more mechanisms are involved in the development of drug resistance, including increased drug metabolism; increased efflux of drugs; anticancer drug interactions with other drugs, nutrients, metal ions and other molecules; genetic factors; immunological factors; increased DNA repair capacity, etc. [11,12,13,14].

A major aspect of any new therapeutic approach in cancer and in any other disease is the level of toxicity and the overall risk/benefit assessment of the new proposed therapy in comparison to existing therapies [4]. The damage to normal cells and other toxic side effects of anticancer and other drugs are crucial parameters during anticancer treatments, greatly affecting the overall morbidity and mortality rate in cancer patients. Specificity in cancer cell targeting is also very important and such approaches are expected to have a better therapeutic outcome. In this context, the risk/benefit assessments for most anticancer therapies are not optimal at present and new drugs and drug combinations are required.

Many new investigational anticancer drugs and drug combinations are currently widely used and others are under development, some of which are related to metal ions, chelators and chelator–metal complexes. For example, metal ions such as gallium, chelators such as hydroxyurea and chelator–metal complexes such as platinum complexes are widely used for targeting many different metabolic pathways, including metal metabolism, in relation to many cancer types [15,16,17]. The impact of metals, chelators and chelator–metal complexes in anticancer therapy can be highlighted by the number of cancer patients receiving only platinum complex therapies, which is estimated to be about 50% of the overall number of cancer patients worldwide [17,18].

Metal ions such as iron, copper and zinc are essential for life but also essential for the growth and proliferation of cancer cells, which, in effect, makes them targets for the design of new metal, chelator or chelator–metal complex-related pharmaceuticals for treating cancer [19,20,21,22]. The targets associated with each of the essential metal ions may include key proteins and their membrane receptors; intracellular transport and transit metal ion pools; different organelles and metabolic pathways, genomic, transcriptional and other factors, etc. [8,22,23].

Several major advances have occurred in the last few years in relation to iron metabolism and the identification of a number of new anticancer drug targets, which, in addition to the inhibition of DNA synthesis and of free radical toxicity, involve various new carcinogenic molecules, as well as different biomolecules and metabolic pathways [24,25,26,27,28]. These include, for example, ferroptosis, macrophage anticancer activity effects, hyperferritinemia, hypoxia-inducible factor (HIF), iron-related carcinogenic molecules, toxins found in food, etc. Selective modulation for achieving anticancer effects could be accomplished in most of these cases by adopting new drug-targeting strategies, including chelating drugs with specific properties and physicochemical characteristics [8,22,24,25,26,27,28]. 

Many other different targeting approaches involving chelators could be employed during the interaction of established anticancer drugs such as doxorubicin, bleomycin and hydroxyurea with metal ions, and in particular, iron, which has been associated with a decrease in anticancer activity and an increase in toxicity [22]. Similar interactions and effects on efficacy and toxicity are also expected with platinum complex drugs and theranostic or diagnostic drugs containing different metal ions [22].

## 2. Targeting Aspects of Chelator Interactions with Iron

Several metal ions are essential for life and widely distributed in all organisms, mainly as vital components of hundreds of proteins; they play very important roles in living systems including in growth, development, energy transactions, oxygen transport, DNA synthesis and genomic and transcription factors. 

**Figure 1:**
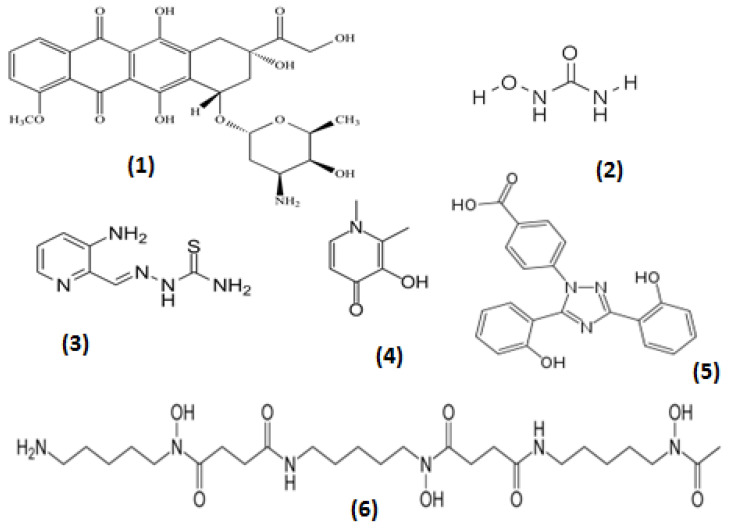
The chemical structure of anticancer drugs and chelating drugs with high affinity for iron. The anticancer drugs doxorubicin (**1**) and hydroxyurea (**2**) are widely used in cancer patients, triapine (**3**) is undergoing clinical trials in cancer and the iron-chelating drugs deferiprone (L1) (**4**), deferasirox (**5**) and deferoxamine (**6**) are widely used in iron-overloaded patients for the removal of excess iron.

In biological systems, all metal ions are bound to ligands containing atoms with a lone pair of electrons, e.g., O, N, S, which can form a bond with the metal ion. Chelators (chele, Greek χειλή—claw of a crab) are organic molecules possessing at least two binding groups, which have high affinity and can bind/carry metal ions. Examples of natural chelators containing iron are myoglobin, hemoglobin, transferrin, phytochelators and microbial siderophores [8,22,26,29,30,31,32,33,34]. Many other natural, dietary and pharmaceutical compounds have iron and other metal-chelating properties and metal interactions, including citrate, ascorbate, folate, ATP, doxorubicin, tetracyclines, hydroxyurea, etc. (Figure 1) [8,22,26,35,36]. 

All chelators and chelating drugs have different physicochemical, biological, pharmacological and toxicological properties including different affinities for metal ions, variable forms of interactions with proteins and different levels of access to subcellular and cellular compartments, cells, tissues and organs (Table 1) [22,34].

The essential metal ions are strictly controlled in biological systems by specific metal-uptake, distribution and excretion metabolic pathways [22]. The controlled acquisition of daily dietary requirements and the maintenance of a specific range of concentrations of metal ions in cells and tissues ensure normal daily physiological activities and bodily functions. Zinc, for example, is required for the turnover of more than 300 catalytically active zinc metalloproteins and more than 2000 zinc-dependent transcription factors [19,20,37].

Iron is one of the most important essential metal ions in humans. Iron metabolic imbalance is associated with many clinical conditions such as iron deficiency anemia, which affects about a third to a quarter of the world’s population [38,39,40,41]. Hundreds of thousands of other categories of patients are also affected by iron overload toxicity due to regular red blood cell transfusions, which mainly involve hematological and malignant diseases including cancer, hematopoietic stem cell transplantation, aplastic anemia, sickle cell anemia and thalassemia [42,43,44,45,46,47,48]. Several other conditions of iron and copper overload also exist as a result of increased gastrointestinal absorption, such as of iron in idiopathic hemochromatosis and copper in Wilson’s disease [21,49,50]. Chronic metal overload in the latter two diseases of increased metal absorption could eventually lead to metal overload, serious toxicities and fatalities, including many cases of hepatocellular carcinoma [21,50,51,52]. 

In general, the accumulation of excess essential and xenobiotic metal ions including iron in any organ, as well as in cellular and sub-cellular compartments, is a negative prognostic factor for any disease. Chelating drugs in many instances of metal overload and other diseases of metal metabolic imbalance could potentially restore physiological balance or modulate metal function and treat the associated diseases [22]. In particular, the chelating drugs deferoxamine, deferiprone (L1), deferasirox and their combinations are widely used daily for the treatment of iron overload in thalassemia and other iron-overloading conditions (Figure 1) [34,53]. Different chelating drugs are used for the detoxification of many other metals [54]. In contrast, lipophilic and other iron complex formulations can be used for increasing iron absorption, e.g., the tris maltol iron complex (ferric maltol or Feraccru or Accrufer) [55]. 

The modulation of the redox activity of iron, iron-containing protein turnover and function, and many associated metabolic pathways in normal and disease states, including cancer, are some of the major targets accessible for therapeutic intervention using iron-chelating drugs and other chelators. Similar targeting prospects can be identified from chelator intervention in the specific cellular activity and function of associated diseases. In this context, the identification and modulation of such targets may have a major impact on the treatment of cancer and many other diseases [22]. 

### 2.1. Molecular Effects of Chelators, Metal Ions and Metal–Chelator Complexes in Anticancer Targeting 

The importance of the benefits and risks of metal ions and metal complexes in cancer therapy has, in general, been overlooked in recent years, despite the majority of cancer patients very often experiencing their therapeutic effects but also their toxicity effects. Full evaluation of their anticancer potential, the related mechanisms involved and their targeting effects are still the subject of pending investigations.

The impact of metal complexes in anticancer therapy is highlighted by the wide use of platinum complex-based drugs. In particular, the platinum complex formulations cisplatin, carboplatin and oxaliplatin are the most common and widely used drugs in different types of cancer [17,18,56,57,58]. Their general mode of anticancer activity and targeting effects are considered to involve DNA. It appears that platinum complexes cause crosslinking of DNA, which subsequently leads to the inhibition of DNA synthesis and also of DNA repair in cancer cells. Each of the drug formulations containing a different platinum-based complex appears to have different pharmacological and toxicological effects and selective anticancer specificity for each cancer type. A major drawback of platinum complex-based drugs is the serious toxic side effects and the acquired resistance to anticancer treatments [11,12,13,14,17,18,56,57,58]. In the meantime, new anticancer platinum complex drugs including those with 1-hydroxypyridine-2-thione or omadine, which was discovered in the 1980′s to have anticancer effects, as well as related combinations are undergoing clinical trials and are in different phases of development [17,18,59,60,61,62,63,64]. Similarly, the development of several other metal complexes and formulations including many theranostic metal complexes also show promising anticancer effects [65].

**Figure 2:**
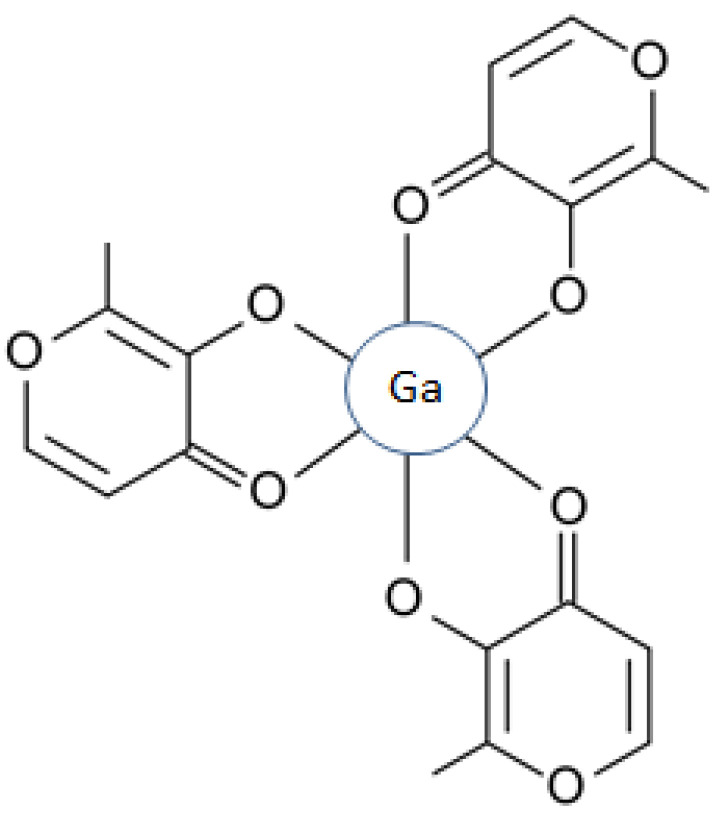
The chemical structure of maltol gallate. Three molecules of maltol are required to form an octahedral complex with one atom of gallium (Ga (III)) at physiological pH. Similar complexes of maltol, and also of deferiprone (L1), are formed with iron and aluminum.

Several other forms of anticancer targeting via the inhibition of DNA synthesis and also via other routes could be achieved using different metal complexes. For example, xenobiotic metal complexes such as maltol gallate are used to transfer gallium intracellularly, which could bind to nucleotide substrates and inhibit DNA synthesis (Figure 2). An additional mechanism of inhibition by gallium and other xenobiotic metals is the disruption of iron and other essential metal metabolic pathways related to DNA synthesis (Figure 2) [15,60].

A rapidly expanding area and targeting application in medicine is the use of diagnostic and theranostic agents in many diseases including cancer. Many of these diagnostic and theranostic agents involve chelators, metals, chelator–metal complexes and metal nanoparticles [60,66,67]. In this context, a plethora of metals are used in cancer and the treatment of other patients, such as gadolinium and technetium in magnetic resonance imaging (MRI); radioactive gallium and indium for radiotracing; radioactive copper, zirconium and scandium in positron emission tomography (PET); metal nanoparticles containing iron, gold, silver, zinc, and titanium, etc. (Figure 2) [60]. 

The design and targeted use of different formulations of chelators, metals, chelator–metal complexes and metal nanoparticles result in different pharmacological activities. In each formulation case, the variable physicochemical properties appear to affect bodily distribution and specificity in tracing and tracking the progress of different types of cancer in the affected tissues (Table 1). Especially in the case of theranostic agents, specificity is evident from the combination of cancer treatment with simultaneous diagnosis and monitoring [60,67,68,69,70]. Metal-containing theranostics have a multifunctional role in cancer targeting and could be used in imaging for diagnosis, radiotherapy, chemotherapy, photo-thermal and other specialized anticancer therapies [60,67,68,69,70]. 

The detoxification of radioactive and other toxic metals following the use of metal theranostic formulations, as well as of metal-based diagnostics, is another major area of clinical interest including concerns regarding the cause of increased cancer incidence following their use [54,60,70,71]. Similar concerns of increased cancer incidence arise from environmental pollution during the use of plutonium, uranium and other radioactive metals in the nuclear industry, as well as heavy metals in other industries [72,73,74,75,76,77,78,79]. In all these cases, the clinical use of specific chelating drugs for toxic metal detoxification can play a major role in cancer prevention. 

### 2.2. Free Radicals and Targeting in Cancer by Chelators, Metal Ions and Metal–Chelator Complexes 

There is a broad diversity in molecular and redox properties related to metal ions, chelating drugs and chelator–metal complexes, leading to a wide range of therapeutic targeting prospects, toxicity issues and other effects in many diseases of free radical pathology, including cancer. In this context, some of the free radical mechanisms and effects observed in cancer can be targeted for clinical applications including the design of anticancer therapeutic strategies [8,22,23,24,25,26,36,80].

Anticancer targeting based on redox activity, including the antioxidant and pro-oxidant interactions of metal ions, chelators and chelator–metal complexes with different types of biomolecules, is a rapidly growing and developing area in cancer therapeutics. In biological systems, redox activity is mostly based on iron and copper complexes, which have variable properties and effects in different normal and cancer cell functions [22,36,80,81,82,83]. Targeting the interactions of proteins possessing iron or copper and also of other proteins involved in the utilization and metabolism of free radicals is important in anticancer strategies. In most of these cases, several other factors may influence the targeting of free radicals and associated anticancer activity at the molecular and cellular levels, such as cell membrane permeation and intracellular transport, access to organelles such as mitochondria and lysosomes, the tumor’s micro-environmental pH and hypoxic state, the presence of reducing agents such as ascorbate (vitamin C), etc. [22,36,80,81,82,83].

**Figure 3:**
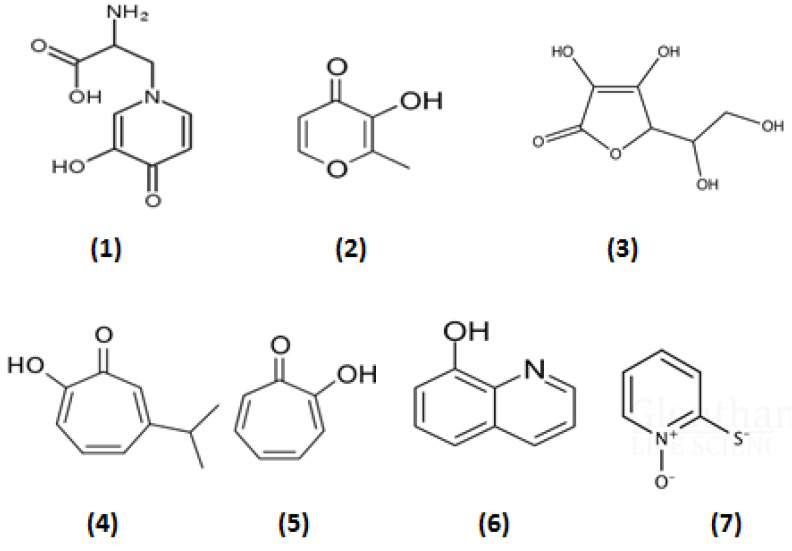
The structure of naturally occurring plant products (phytochelators) with iron-binding and anticancer properties. Mimosine (**1**), maltol (**2**), ascorbic acid (vitamin C) (**3**), isopropyl tropolone (**4**), tropolone (**5**), 8-hydroxyquinoline (**6**), 1-hydroxypyridine-2-thione or omadine (**7**).

There are many examples of different interactions and anticancer targeting prospects for chelators in relation to the iron- and copper-catalyzed production of free radicals and their effects. The redox properties of these metal ions, chelating drugs and their metal complexes, which are used to treat various diseases, are one of the central factors in their therapeutic activity and also a major aspect of their toxicity. Specific mechanisms, targeting approaches and conditions apply in each case [26,80,81,82,83,84,85]. For example, a detailed report discussing the redox properties and mechanistic insights of three major classes of chelating drugs, namely alpha-ketohydroxypyridines (e.g., L1), anthraquinones (e.g., doxorubicin) and thiosemicarbazones (e.g., triapine) and also their metal complexes, were recently reviewed, with emphasis on their biological effects and clinical applications, including potential anticancer activity (Figure 1) [86,87,88,89,90,91,92,93,94,95,96,97,98,99]. The effects and interactions of these and other drugs or naturally occurring plant products (phytochelators) with vitamin C were also discussed, considering that vitamin C is a metal-chelating and antioxidant plant nutrient with major involvement in redox reactions, and also the subject of many clinical trials as an anticancer agent in various types of cancer (Figure 3) [26,86,100,101,102,103,104,105,106].

In most cases, specific chelators with a high affinity for iron and/or copper can be used to inhibit the catalytic and redox activity of each metal ion, as well as associated oxidative stress processes related to cancer growth and proliferation [26,100,101,102,107,108]. In particular, the iron-chelating drugs L1, deferasirox and deferoxamine can bind iron and inhibit the associated free radical reactions involved in such processes [26,100,101,102,107,108]. Deferiprone has also been shown to inhibit free radical reactions catalyzed by copper ions [109]. 

Conversely, in some forms of cancer cell targeting, metal complexes are required, which have redox activity and can enhance the redox properties of bound metal ions for anticancer activity. The anticancer targeting potential of chelator–iron complexes was first identified in the 1980s using the lipophilic iron complexes of omadine, tropolone and 8-hydroxyquinoline, which exhibited potent anticancer activity much higher than that of their metal unbound forms and in comparison to hydrophilic chelators such as L1 and deferoxamine (Figure 1 and Figure 3) [59,60,110]. Similarly, triapine and other thiosemicarbazones are also highly lipophilic; however, they also appear to have higher affinity for ferrous iron than ferric iron and their iron complexes appear to be redox active, which is not observed in the case of L1, deferoxamine and deferasirox [111,112,113]. The anticancer activity of triapine and other thiosemicarbazones is thought to be partly related to the inhibition of DNA synthesis via the inhibition of ribonucleotide reductase [114,115]. The copper and zinc complexes of triapine and other thiosemicarbazones have also been shown to have anticancer potential (Figure 1) [116,117]. 

In the anthraquinone group, some of which are anthracycline antibiotics with a quinone metal-chelating site, have been known to have anticancer activity. In particular, doxorubicin, daunorubicin and emodin are widely used anticancer drugs in, for example, breast cancer, lymphoma and leukemia. The mode of action of anthraquinones includes cytotoxic effects, which lead to the inhibition of topoisomerase II and disrupt DNA synthesis. Furthermore, anthraquinones undergo redox cyclic reactions, which are accompanied by the release of reactive oxygen species (ROS) in biological electron transfer systems. However, the free radical activity is also considered responsible for the cardiotoxic effect inherent in this class of compounds. Other toxicities include low selectivity for cancer cells, hair follicle toxicity, etc. [86,92,93,94].

Overall, the spectrum of chelator mechanisms that affect the redox activity of complexed metals is quite diverse and includes different parameters for each target. Among these parameters is the ability of the chelator to participate in redox reactions, a change in the electrode potential of the metal in the complex, the creation/preservation of the coordination center for metal binding in the complex, the surrounding pH of the target, the presence of other reducing or oxidizing agents and the possibility of mixed complexes with other chelators [86]. 

Despite that redox activity plays a major role in anticancer targeting strategies involving chelators, iron and other metal ions, as well as related metal–chelator complexes, the same activity is also observed in the mode of action and toxicity of widely used anticancer drugs such as doxorubicin and hydroxyurea. In this context, redox activity should be considered a major aspect of screening for targeting not only for all anticancer drugs but also for the toxicity of new investigational anticancer drugs. The inhibition of free radical toxicity by effective iron and copper chelators such as L1 supports their selective use as main or adjuvant antioxidant therapies in cancer and other diseases of free radical pathology [22,26,80].

Future possibilities for new therapeutics can be envisaged by designing new investigational chelating drugs; this includes the modification of chemical structures by changing their functional groups and other substituents, which can change their affinity for different metal ions, their redox potential, their partition coefficients and other properties, all of which could potentially lead to differential and more specific targeting in cancer. Furthermore, improved anticancer strategies could be designed, such as combination therapies with other anticancer drugs or redox-active drugs and/or natural products including vitamin C and mimosine, which could lead to improved anticancer targeting. 

### 2.3. Ferroptosis, the New Target in Cancer Therapies and the Role of Chelators 

The importance of iron in health and disease, and especially in cancer mechanisms, has been further highlighted by the recent discovery of ferroptosis, which is a form of oxytosis caused by free radicals arising from iron and involves transcription and many other factors [118]. In addition to cancer, ferroptosis is implicated in infectious, neurodegenerative, cardiac, kidney and many other diseases including COVID-19 [119,120,121,122,123,124]. There have been more than 2000 publications in the last 10 years describing many different aspects of the role of ferroptosis in cancer, with the annual number of citations and publications growing exponentially over the past decade [125].

Under normal conditions, cell death is an essential process for the controlled development and maintenance of homeostasis, and also for the prevention of proliferative diseases, including cancer. Cancer cells can undergo several forms of regulated cell death, such as apoptosis, during tumor development, and the activation of apoptosis or ferroptosis is considered a primary and promising strategy for cancer therapy [118].

Ferroptosis is a cell death program which is morphologically, biochemically and genetically distinct from apoptotic and necrotic cell death and is mainly characterized by an increase in intracellular iron, the sustainable production of increased free radical reactions caused mainly by labile and other forms of iron, a decrease in glutathione production, the inhibition of glutathione peroxidase (GPX4) activity, the accumulation of lipid peroxides on cell membranes, and an iron-dependent autophagic cell death program [126]. 

Many areas are under investigation regarding ferroptosis and cancer, with all the different aspects and parameters involved covered; these include the mechanisms of different cancer cell types and stages, the role of iron and lipid metabolism, the effects of drugs and nutrients, the prospect of anticancer targeting and the design of new therapeutic strategies. In particular, the conditions and factors involved at each stage of ferroptosis in cancer need full evaluation before any therapeutic intervention. In this context, the possible development and application of chelating drugs in some of the anticancer targets related to ferroptosis could be considered at the molecular, cellular and clinical level, preferably in cancer cell types for which existing therapies are ineffective, and also in cases of cancer metastasis and drug resistance, where overall survival in cancer patients is very low.

There are many targeting options regarding therapeutic strategy interventions in ferroptosis in relation to cancer, including the targeting of associated proteins, transcription factors, iron metabolic pathways, lipid metabolic pathways, free radical generation and associated metabolic pathways, redox iron, ferritinophagy, macrophage-stored iron and many others. In most of these cases, iron-chelating drugs and other drugs with chelating properties, as well as chelator–iron complexes, can play a major, adjuvant or synergistic role in optimizing anticancer therapeutic applications. Indeed, in the initial cell studies of the discovery of ferroptosis, lipophilic antioxidants such as vitamin E and hydrophilic chelators such as deferoxamine were shown to inhibit ferroptosis [126,127]. 

Targeting ferroptosis in cancer is a complex issue involving different conditions in the tumor microenvironment; sometimes, opposing mechanisms may lead to different outcomes such as the activation of ferroptosis, which can result in the nonapoptotic destruction of certain types of cancer cells. Similarly, some other factors and mechanisms may promote or inhibit ferroptosis, affecting, among other things, tumor suppression and tumor immunity, the diverse susceptibilities of cancer cell types at different cancer cell stages, etc. [126,127,128,129,130]. 

Cancer progression is facilitated by conditions developing in the tumor microenvironment where, for example, hypoxia contributes to cancer cell invasion, metastasis and therapy-resistant phenotypes. In this case, iron is considered a promoter of proliferation of hypoxic cancer cells through mechanisms of enhancement of iron utilization and storage, as well as efflux through exosomes, which further contributes to cancer cell proliferation, metastasis, ferroptosis resistance and immune escape [129,130,131]. In contrast, the surrounding normal stromal cells in the hypoxic and iron-deficient microenvironment developed by tumors become more weakened and more susceptible to destruction by tumor cells due to iron deficiency, facilitating further cancer cell proliferation. 

One of the major characteristics of the ferroptotic process is the accelerated cell damage caused by the increased iron release originating mostly from ferritinophagy, an autophagic process which involves the iron-storage protein ferritin. Under normal conditions, ferritinophagy involves the sequestration of ferritin into autophagosomes and its delivery to lysosomes for degradation, resulting in the release of ferritin iron [132,133,134]. It is envisaged that for the purpose of anticancer targeting, different factors can influence the process of autophagy and lysosomal degradation, as well as affect ferritinophagy and ferroptosis and the overall rate of proliferation of cancer cells [132,133,134,135]. In this context, and considering the various targets associated with ferroptosis, iron-chelating drugs mobilizing transferrin and ferritin iron as well as iron released during ferritinophagy could, in principle, be used as main, adjuvant or synergistic therapies for reducing or inhibiting cancer cell proliferation, metastasis and immune escape. Similar effects on ferroptotic cell death may be suggested by chelator inhibition of the iron-containing enzymes cyclooxygenase and lipoxygenase, which are involved in lipid peroxidation during arachidonic acid metabolism. Many chelators, including the chelating drugs L1 and deferoxamine, have been previously shown to inhibit cyclooxygenase and lipoxygenase, especially when present at high concentrations [136,137].

Another major area of anticancer drug development in relation to ferroptosis is the identification of drugs or factors for use in cancer cell types resistant to existing drug treatments and also to ferroptosis. In this case, several approaches have been suggested for inducing ferroptotic cell death, including gene modulation associated with increasing intracellular iron uptake, increased lipid levels and the disruption of lipid peroxidation repair, and the modulation of protein functions associated with the aforementioned genes. However, and most importantly, simple molecules such as the naturally occurring iron phytochelator gallic acid and brucine have also been identified to induce ferroptotic cell death in resistant cancer cell types [26,138]. 

The anticancer properties of gallic acid have suggested basic mechanisms of the intracellular incorporation of iron (III) and cancer cell iron overload, reduction to iron (II), the increased production of reactive oxygen species (ROS) and the induction of ferroptosis [138]. The same mechanisms were observed more than 30 years ago with the naturally occurring anticancer iron phytochelators omadine, hydroxyquinoline and tropolone and their iron complexes, which caused iron overload in cells and cancer cell death at very low concentrations [59,110,139]. Overall, lipophilic chelators appear to cause iron overload by transporting iron intracellularly and by maintaining iron intracellularly, as well as reversing or reducing the efflux of iron during ferritinophagy [140,141]. Furthermore, the presence of vitamin C is likely to speed up ferroptosis in resistant cancer cells because of its ability to reduce iron (III) to iron (II). Further studies are needed to clarify the role of lipophilic and other chelators and reducing agents in the ferroptotic cell death process of cancer cells [142]. 

Several other prospects for anticancer targeting in relation to ferroptosis could be considered using specific iron chelators and chelator–iron complexes. For example, combination therapies of chelators or chelators with other anticancer agents could be used in initiating ferroptosis in relation to drug resistance to established therapies. In these cases, the regulation of ferroptosis appears to offer therapeutic solutions for refractory and recurring tumors in almost all types of cancer, metastasis and drug resistance [143,144,145,146,147,148,149,150,151]. In other cases, such as in therapeutic combination with immunotherapy, ferroptosis can be enhanced and improve the efficacy of immunotherapy [152,153,154]. 

Many other pharmacological and other factors should be considered in the use of chelators and chelating drugs in ferroptosis. For example, the mode of action of specific drugs should also be considered for targeting ferroptosis in difficult-to-access tumors, e.g., L1 could be selected and used in special cases of ferroptosis such as glioblastomas because of its specific pharmacological properties, including its ability to cross the blood–brain barrier, which is not a property shared by other iron-chelating drugs [138,155]. Further considerations in relation to targeting include many other parameters such as pharmacokinetics, e.g., the duration of action and concentration of the drug in comparison to the duration of ferroptosis and the rate of formation to free radical production and lipid peroxidation [156,157]. 

### 2.4. Targeting of Iron-Laden Macrophages and Hyperferritinemia by Chelators in Cancer

Macrophages are white blood cells, which originate from hemopoietic stem cells of the bone marrow and differentiate into monocytes, which are then released into the bloodstream. They play a major role in the immune system and immune response, performing many functions including phagocytosis, which can result in the elimination of foreign entities/pathogens including foreign substances, cellular debris from damaged or aging cells, microbes and cancer cells. Macrophages associated with tumors are important components of both the immune system and the tumor microenvironment, and interact with ferroptosis and other pathways involved in cancer initiation, proliferation, metastasis, drug resistance, etc. [158,159,160,161,162]. 

Macrophages also play a key role in innate immunity and are constituents of the reticuloendothelial system; they occur in almost all tissues of the body under different names such as monocytes in the bone marrow and blood, Kuppfer cells in the liver, microglia in the central nervous system and osteoclasts in bone and alveolar macrophages in the lungs. In general, macrophages are involved in the detection of diseases as a part of the immune response and used as biomarkers, including in most cancer cases. In this context, assessing and characterizing macrophage infiltration in tumor biopsies or tissues provides a prognostic assessment, which, in general, is correlated with cancer progression, outcomes and therapeutic responses [140,163,164,165]. 

Under normal conditions, cancer cells are recognized by the immune system and removed by macrophages. However, it appears that in many cases, such as in the aged human population where innate immunity declines with age, there appears to be, among other changes, immune evasion and the escape of immune surveillance by many pathogenic cells including cancer cells; this leads to the multiplication of cancer cells and, progressively, to the formation and development of tumors [166,167,168]. Furthermore, it appears that local immunosuppression can be developed in the tumor microenvironment under hypoxic and acidic conditions in which cancer cells utilize adjacent endothelial and other cells for energy supply and nutrients, including iron, which mostly originates from ferritin [24,169]. Maximum iron release is facilitated during ferritinophagy under acidic conditions, which is then effluxed by cancer cells and taken by macrophages, causing the formation of iron-laden macrophages [133,134,135,170,171,172]. Furthermore, and in addition to ferritinophagy, the uptake of increased amounts of iron by macrophages can also be accomplished via transferrin receptors, erythrophagocytosis, hemoglobin catabolism and damaged cells.

The presence of iron-laden macrophages in the tumor microenvironment is not only of prognostic value but is also a major functional drawback in the ability of macrophages to act against cancer cells. The higher the level of iron loading in iron-laden macrophages, the lower their ability to act unilaterally or with other therapeutic interventions against cancer cells. 

Another feature of iron metabolic changes observed in cancer, in association with the presence of iron-laden macrophages, is the substantial increase in serum ferritin levels, called “hyperferritinemia”. Similar to the presence of increased levels of iron-laden macrophages, hyperferritinemia is a negative prognostic factor for any disease, including cancer, and it can also be a result of different forms of inflammation, autoimmune disorders and infection [173,174,175,176,177,178]. The immune response in all these conditions, including cancer, leads to the activation of macrophages, iron uptake into macrophages and a reduction in serum iron, with concurrent increased synthesis and secretion of ferritin in the plasma by macrophages [170,171,172,173,174,175,176,177,178]. Reduced normal function, including the reduced anticancer, antimicrobial and other activity of iron-laden macrophages, progressively leads to increased cancer cell proliferation. In contrast, the increased iron uptake in macrophages and the reduction in serum iron result in iron deficiency in adjacent normal cells, where the concomitant reduction in iron-dependent enzymatic activity in many iron metabolic pathways can cause a general reduction in normal cell functions including apoptosis [179]. 

The reduction in the anticancer activity of iron-laden macrophages, as well as the associated iron deficiency in normal cells, can be restored by iron chelators that can remove the iron load from macrophages. In particular, L1 is the most effective drug for iron mobilization from ferritin and hemosiderin and it has also been shown to remove iron from macrophages and many other cells types in an iron mobilization process that appears to be chelator-concentration-dependent [180,181,182]. 

Most importantly, L1 and L1’s combination with deferoxamine have been shown in clinical trials and in clinical practice to completely clear excess deposited iron from the Kuppfer cells of the liver and iron-laden macrophages of the spleen in thalassemia patients [183,184,185,186,187,188]. Similar effects are expected in regularly red-blood-cell-transfused iron-loaded or non-transfused cancer patients. It is anticipated that in the latter case, complete iron removal from iron-laden macrophages using the above chelation protocols could be accomplished in about 1−2 months [183,184,186].

Similarly, L1 has also been shown to remove iron from macrophages in the reticuloendothelial system in inflammatory diseases such as anemic rheumatoid arthritis and, subsequently, cause an increase in the hemoglobin levels of the patients [189,190]. This has been accomplished via the removal of excess iron by L1 from iron-laden macrophages in the reticuloendothelial system; the exchange of iron with transferrin; and the increase of transferrin saturation with subsequent donation of iron to the erythropoietic cells for the production of hemoglobin, and also to other cells with reduced iron stores [189,190].

The mode of action of specific drugs in relation to their clinical application should always be considered in the context of their specific pharmacological and toxicological properties. For example, L1 could be used in special cases of brain cancer such as glioblastomas because of L1’s ability to cross the blood–brain barrier, which is not a property shared by other iron-chelating drugs [34,53,80,155]. The ability of L1 to remove excess deposited iron detected in the brains of patients has been established using magnetic resonance imaging (MRI T2*). A reduction in excess stored iron in the brain monitored by MRI and accompanied clinical improvement has been shown in different categories of patients, including those with Friedreich’s ataxia, Parkinson’s disease, pantothenate kinase-associated neurodegeneration (PKAN) and neurodegeneration with brain iron accumulation [191,192,193,194]. It is anticipated that similar effects could be achieved with patients suffering from brain cancers and who have increased iron accumulation in their macrophages. 

Several other proposals could be considered in anticancer targeting in relation to iron-laden macrophage modulation. For example, the introduction of iron chelation therapy, preferably at the early stages following cancer diagnosis, could both reduce the iron efflux effects of cancer cells and increase the anticancer activity of macrophages and other related therapeutic interventions. Similarly, the interaction of chelating drugs with immunological and other drugs used by cancer patients should also be considered for optimizing related cancer therapies.

## 3. Protein Interactions of Chelators and Chelator–Iron Complexes in Anticancer Targeting

The major role of proteins related to iron metabolism and associated pathways in relation to anticancer targeting has already been mentioned in several of the previous sections. In particular, key proteins related to iron and to the growth, proliferation and metastasis of cancer cells include ribonucleotide reductase, which plays a major role in DNA synthesis; transferrin for iron transport in the blood; transferrin receptors for regulating iron intake intracellularly; ferritin for intracellular iron storage and utilization; and many others [8,9,10,22,23,24]. Furthermore, there are proteins involved in ferroptotic cell death, which include ferritin for the release of stored iron, iron proteins involved in lipid metabolism such as lipoxygenase and cyclooxygenase, proteins such as aconitase involved in the tricarboxylic acid cycle and glucose metabolism, proteins involved in hypoxia control and proteins involved in the control of redox activity caused by iron, as well as many others [22]. It could be envisaged that all of these proteins and their associated pathways constitute targets and they could all be targeted and used for the design of anticancer therapeutics. In particular, a major role for such anticancer targeting strategies could involve specific iron-chelating drugs and chelator–metal complexes which can modulate associated iron metabolic pathways [34].

The interactions of chelators and chelator–iron complexes with proteins of iron metabolism differ in each case and depend on many physicochemical, pharmacological and other parameters (Table 1) [22,34]. In considering anticancer targeting, the interactions with proteins can be related to direct or indirect effects. In the latter case, chelators could, in general, inhibit the activity of iron-containing proteins by removing or withholding iron bound to transferrin, “transit” iron at the intracellular iron pool and iron in ferritin, thus slowing down the turnover of iron-dependent proteins and related metabolic processes [8,22,34]. There are many other chelator-specific interactions with proteins related to anticancer effects, which may also have implications in future therapeutic strategies against cancer. 

The mode of action of chelators in relation to iron release from ferritin in ferroptosis and iron-laden macrophages, as well as associated metabolic pathways, is of major interest for anticancer targeting. The mobilization of ferritin iron before, during and after ferritinophagy in ferroptotic cell death could reduce the rate of cancer cell proliferation. Considering the target characteristics and, in particular, the iron forms present in iron-laden macrophages, this is predominantly in the form of hemosiderin, a breakdown product of ferritin without the whole protein shell but of similar oxohydroxide polynuclear iron complex composition [164,181,183,184,185,186,187,188]. Ferritin, found in all cells and also in serum, can potentially store up to 4500 molecules of iron in an oxohydroxide polynuclear iron complex and is considered a major target for iron chelation. In contrast, serum ferritin, which mostly originates from macrophages, is used as a diagnostic tool for body iron stores and it increases in iron-overloading diseases and decreases in iron deficiency anemia [170,171,172,173,174,195]. Serum ferritin contains negligible amounts of iron and cannot be considered a target for iron mobilization. However, ferritin can also play many other roles in addition to storing iron, including that of a signaling molecule, a modulator of the immune response, an acute phase reactant, as well as a regulator of cytokine synthesis and release [171,172,173,174,175,176,177,178]. In this context, many of the above processes are expected to be affected during the use of chelation therapy for anticancer targeting.

The mode of action and rate of iron mobilization from ferritin and hemosiderin by chelators has been previously reviewed [22]. Overall, it differs between chelators but is generally slow, and it may take several days to reach completion, with only a portion of the iron stored in the proteins removed [180,181,196]. Many other factors can also influence the rate of iron mobilization from ferritin and hemosiderin by chelators, which, by comparison, is faster from freshly formed polynuclear iron precipitates than hemosiderin and much slower from ferritin [22,180,181,196]. In general, the amount of iron removed by chelators in vitro depends on the concentration of the chelators and the quantity of iron stored in the proteins [22,180,181,196]. In particular, the rapid intracellular transfer of L1 and its efficacy in ferritin iron mobilization may have advantages over other chelating drugs. Similar findings of iron mobilization have also been observed during the chelation treatment of iron-loaded patients and are also expected in the case of iron removal from iron-laden macrophages in cancer patients, where the higher the chelating drug dose and iron load of the patient, the more iron is mobilized and excreted [183,184,185,186]. 

**Figure 4:**
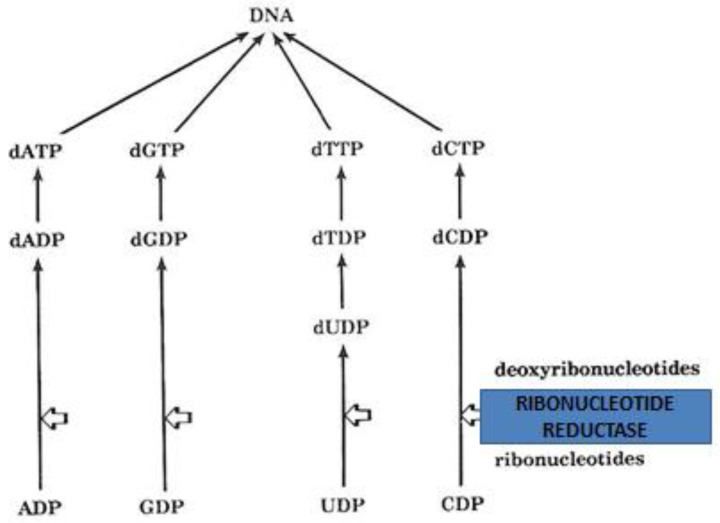
The mode and site of action of the iron-containing enzyme ribonucleotide reductase, which is essential for DNA synthesis. Ribonucleotide reductase is responsible for the conversion of ribonucleotides (ADP: adenosine diphosphate; GDP: guanosine diphosphate; UDP: uridine diphosphate; and CDP: cytidine diphosphate) to deoxyribonucleotides (dADP: deoxyadenosine diphosphate; dGDP: deoxyguanosine diphosphate; dUPD: deoxyuridine diphosphate and dCDP: deoxycytidine diphosphate. The nucleotides dATP, dGTP, dTTP and dCTP (deoxy nitrogen-based triphosphates) are then formed in cells and used for DNA synthesis.

Of major interest in anticancer targeting and the design of anticancer strategies is the allosteric interaction of the widely used anticancer chelating drug hydroxyurea with ribonucleotide reductase, the key iron-containing enzyme needed for DNA synthesis (Figure 4). The mechanism of action of the anticancer drug hydroxyurea is thought to involve the inhibition of ribonucleotide reductase through the free radical nitroxide metabolite of hydroxyurea, which quenches the tyrosyl free radical at the active site of the M2 protein subunit of the enzyme [16,114,197,198]. In addition to anticancer therapy, hydroxyurea has other clinical effects and is also used in the treatment of other diseases such as sickle cell anemia and thalassemia intermedia, where it reduces the polymerization of sickle hemoglobin and reduces the level of red blood cell transfusions due to increases in fetal hemoglobin production, respectively (Figure 1) [199,200,201,202,203,204,205,206]. Hydroxyurea has recently been identified as a natural product with possible antiviral and other applications [207,208,209]. Several hydroxyurea analogs have also been synthesized and tested as anticancer agents [210]. Chelating drugs such as deferoxamine and L1 have also been shown to inhibit ribonucleotide reductase and DNA synthesis in several cancer cell lines but at higher concentrations than hydroxyurea (Figure 1 and Figure 4) [22,139,211].

Drug interactions with transferrin and the control of iron delivery to cancer cells is another major target for anticancer drug design involving all cancer stages including initiation, proliferation and angiogenesis [22,212,213]. In general, there is wide variation in the iron requirements for different cell types, which are mainly reflected by the number of transferrin receptors on the cell membrane [22,214]. In this context, increased transferrin receptors are present in different types of normal cells, e.g., hepatocytes and erythropoietic cells in comparison to other normal cells. Similarly, increased transferrin receptors are present in cancer cell types such as breast, prostate and bladder, and in leukemia, in comparison to other cancer cell types [8,215,216]. The control of the delivery of transferrin iron and other forms of iron to cells by chelators, and also through the modulation of transferrin receptors, can affect key enzymes and other organic biomolecules in cancer cells and may have an application in the design of specific anticancer therapeutic strategies [8,212,213,214,215]. 

Several other mechanisms of anticancer therapeutic strategies involving the reduction or prevention of iron intake in cancer cells may also be considered through the delivery of other metals closely related to iron, such as gallium, by transferrin [214,217]. Chelating drugs such as L1, which affects iron removal and gallium delivery to transferrin at different concentrations, could also be used in such targeting methods [214,217,218,219]. A different anticancer therapeutic strategy may involve the targeting of cancer cells using anticancer drugs with metal-binding properties, such as doxorubicin and bleomycin, some of which are capable of forming stable ternary iron complexes with transferrin, similar to iron maltol, which could be transferred and incorporated by transferrin into cancer cells [87,220]. The specificity of targeting in relation to the delivery of such drugs would depend on the presence of an increased number of transferrin receptors, which are mainly present on selected cancer cell types. The use of antibodies and other interventions against transferrin receptors is another rapidly growing area of anticancer targeting [221,222,223]. 

A combination of different anticancer interventions including chelation and transferrin, lactoferrin and other forms of iron deprivation or replacement by other metal ions, such as those discussed above, may be another anticancer therapeutic strategy mechanism for most cancer cell types [212,214,224,225,226]. 

Targeting hypoxia in the tumor microenvironment is another strategy for the design of anticancer therapeutics and for preventing, in general, the onset and progression of cancer [227,228]. Similarly, reversing or reducing hypoxia in the tumor microenvironment is an effective strategy to improve the therapeutic performance of immunological and other drugs [227,228]. In particular, drugs activating the anti-hypoxic response at the cellular level may improve the hypoxic state in the tumor microenvironment and decrease cancer cell growth and proliferation. In this context, the identification of hypoxia-inducible factor (HIF), a transcription factor which switches the cell from aerobic to anaerobic glycolysis, is considered to play a key role in hypoxia and to be a possible target for anticancer drugs [229,230]. HIF is a substrate of HIF prolyl hydroxylase (HIF PHD), a non-heme iron-containing dioxygenase which, among other functions, activates a group of genes participating in glucose metabolism. Hydroxylation appears to be the main regulator of HIFα subunit protein stability, which is controlled by HIF PHD [231,232,233,234,235,236]. One of the major targeting prospects of iron chelators, and especially L1, in relation to hypoxia in cancer is their ability to inhibit HIF PHD [236,237,238]. In general, but also in many other cases of iron-containing enzymes, iron removal or displacement by chelators can reduce the activity of HIF PHD and increase the anti-hypoxic response, thus reversing or reducing the hypoxic state in the tumor microenvironment and inhibiting overall cancer cell proliferation [22].

In addition to their anti-hypoxic response, HIF PHD chelator inhibitors have potential for the treatment of anemia, which is prevalent in many cancer patients. This is achieved through a mechanism of enhancement in the production of endogenous erythropoietin and the overall increase in erythropoiesis [189,190,233,234]. Many cancer patients receive a large number of red blood cell transfusions, causing an accumulation of excess iron in the body with negative prognosis [239]. Hydroxylases are also involved in a variety of other functions where the regulation of iron and other co-factors such as vitamin C are important parameters in the treatment of associated diseases [100,240]. Effective inhibition of hydroxylases has been observed using L1 and other chelators in in vitro, in vivo and clinical studies, all of which can increase the prospects of their use in anticancer strategies [236,237,238].

A major protein target for anticancer activity is aconitase [22,241,242]. This iron-containing enzyme appears to be sensitive to intra-mitochondrial iron concentration and fluxes, superoxide and hypoxia [241,242,243,244]. The general activity of mitochondrial aconitase involves the catalytic isomerization of citrate to isocitrate in tricarboxylic acid cycle metabolism. The active site of aconitase has an iron–sulfur ([Fe_4_S_4_]^2+^) cluster, where one iron atom is labile and can be displaced when cellular iron levels are low, thus forming the [Fe_3_S_4_]^+^ cluster, which cannot be regenerated, and inactivating the enzyme [22,241,242,243,244]. The iron–sulfur cluster in aconitase is also particularly sensitive to superoxide, which can also inactivate the enzyme. Aconitase activity affects mitochondrial metabolism and function, which is crucial for tumor proliferation, survival and metastasis. In particular, metastatic tumor cells, chemotherapy- and radiotherapy-resistant tumor cells and cancer stem cells rely on mitochondrial respiration and can be affected by specific targeting of aconitase [243,244,245,246,247].

Several chelators including iron-chelating drugs and natural phytochelators have been shown to inhibit aconitase activity, mitochondrial function and cancer cell growth. This was accomplished by several pathways related to chelation activity including depletion or inhibition of the transfer of mitochondrial iron, an increase in superoxide production, the inhibition of hypoxia and possibly other associated factors or enzymes [248,249]. In this context, of particular interest is L1 and mimosine, which can enter mitochondria and affect several of these processes (Figure 1 and Figure 3) [248,249]. Despite L1 and mimosine being some of the most potent antioxidant drugs, e.g., in inhibiting the Fenton reaction, related to iron and copper toxicities, they can also exhibit pro-oxidant activity under certain conditions. In this context, it appears that L1 and mimosine react with iron (II) in iron–sulfur clusters and mediate the transfer of electrons to oxygen with the formation of superoxide, which inactivates aconitase [86,248,249]. The pro-oxidant activity of L1 and mimosine on aconitase and the general mitochondrial metabolism changes have been proposed as major anticancer mechanisms and additional pathways against cancer cells. 

There are many other proteins, genes and factors related to mitochondrial iron metabolism and ferroptosis, as well as associated pathways, which appear to play an important role in all the stages of cancer development including initiation, proliferation and metastasis [250,251,252,253,254,255,256,257,258]. Similarly, there are a large number of chelators and chelator–metal complexes which could potentially influence all the related pathways and stages of cancer and be used as anticancer agents. 

## 4. Clinical and Other Recent Effects of Iron-Chelating Drugs as Cancer Therapeutics

Many efforts have been undertaken by national and international health authorities in the prevention and early detection of cancer, which can decrease the associated high rate of morbidity and mortality observed in many types of cancer worldwide including prostate and breast cancers, primarily affecting men and women, respectively. The introduction of new anticancer drugs with specific targeting characteristics is imminent because of variations in the response to current treatments, including low efficacy, high toxicity and drug resistance in many subgroup cancer patient populations.

A large number of anticancer agents are continuously being tested for the introduction of new promising therapeutics in different types and stages of cancer. Many of these have reached the stage of clinical trials, including iron-chelating drugs, all of which have to be selected based on a risk/benefit assessment in comparison to anticancer treatments currently available for each specific cancer type [4,5,6,60]. 

The main iron-chelating drugs, L1, deferoxamine and deferasirox, are regularly and widely used in multi-transfused cancer patients for the removal of excess iron, which is a general negative prognostic factor for all types of cancer and other categories of patients (Figure 1) [9,38,46,47,239]. 

In the meantime, several other widely used anticancer drugs including doxorubicin, daunorubicin, emodin and bleomycin are known to chelate iron, but their anticancer activity is not considered to be through iron modulation [8,86,95,96]. However, some of their toxic side effects, observed in many thousands of cancer patients, are thought to involve iron, especially in the case of doxorubicin and other anthracyclines, which cause cardiotoxicity [8,86]. The chelator prodrug dexrazoxane (cardioxane or ICRF 187) is used mainly to chelate iron and to reduce cardiotoxicity caused by doxorubicin and other drugs [259,260]. Similar reversal effects of doxorubicin cardiotoxicity have been shown by L1, which has shown cardioprotective effects in many clinical and non-clinical studies [261]. The cardiospecific iron removal effects of L1 are based on several properties, such as its accessibility to toxic iron pools including hemosiderin and ferritin deposits present in myocytes, the high concentrations that can be achieved following its oral administration, the rapid clearance of its iron complex from myocytes and the body in general, and its ability to minimize the accumulation of excess iron in the heart [183,184,185,186,262]. 

Despite the wide use of iron-chelating drugs in iron loaded cancer patients, their anticancer potential has not yet been fully explored or evaluated. It appears that, in general, only in the case of hydroxyurea treatment in different categories of cancer patients is a mechanism for the modulation of iron function in ribonucleotide reductase used for anticancer effects (Figure 4) [16,210]. However, several clinical studies on chelating drugs and experimental chelators in cancer patients have been carried out over the years and some show encouraging results [213,263,264,265,266]. For example, deferoxamine has been used in combination with other anticancer drugs in neuroblastoma patients and also in leukemia patients [267]. Similarly, the iron- and copper-chelating drug triapine has been tested in phase II clinical trials, in combination with other anticancer drugs and in different formulations (Figure 1) [64,98,99,268,269].

In recent studies involving specialized screening methods with major clinical implications in anticancer targeting, L1 has been shown, at clinically relevant concentrations, to exhibit potent anticancer activity in prostate and breast cancer models, suggesting that there are possibilities for its repurposed use in these and other groups of cancer patients [270,271]. In particular, in a prostate cell model system (TRAMP-C2), L1 at 0.1 mM caused a significant reduction in cell migration, a decrease in glucose consumption in metastatic cells, decreases in mitochondrial functional parameters associated with the oxygen consumption rate and significantly lower mitochondrial aconitase expression and activity [270]. Similarly, in a cancer stem cell study using breast cancer cell lines (MCF7 and T47D) at the same L1 concentration (0.1 mM), inhibition of cancer stem cell propagation was observed through a decrease in mitochondrial oxygen consumption and glycolysis, as well as an increase in mitochondrial superoxide production, suggesting that L1 is one of the most potent EMA- and FDA-approved drugs for targeting cancer stem cells [271].

Another route of anticancer activity related to iron metabolism and mitochondrial dysfunction targeted by L1 has recently been identified through the inhibition of a protein functioning as a metalloreductase that has the ability to reduce both Fe(III) to Fe(II) and Cu(II) to Cu(I), using NAD(+) as acceptor. This “six-transmembrane epithelial antigen of prostate, family member 4” (STEAP4) protein is encoded by the STEAP4 gene and its expression has been shown to be modulated in response to inflammation, oxidative stress and the metabolism of fatty acids and glucose [138]. Overexpression of STEAP4 has been shown in human colon cancer and androgen-dependent prostate cancer compared to normal colon and prostate. Similar findings of overexpression of STEAP4 have been shown in breast cancer HER2+ (human epidermal growth factor receptor 2) cells, where L1 (0.025–0.05 mM) and the HER2+ inhibitor Lapatinib caused significant inhibition of STEAP4 and significant reduction in breast cancer cell growth [272]. Many other proteins, as well as transcription and other factors, have recently been identified to be related to iron metabolism and associated pathways, including the metastasis suppressor N-MYC downstream-regulated gene-1 (NDRG1) [272,273,274]. It appears that the iron-regulated metastasis suppressor NDRG1 plays a major role in the regulation of oncogenic signaling, which can be modulated by L1, deferoxamine, triapine and other chelators [273,274]. In this context, iron chelators targeting the modulation of NDRG1 and similar biomolecules involved in the regulation of metastasis can play a major role in inhibiting cancer progression and metastasis and increasing overall cancer patient survival. 

These recent promising results, as well as previous anticancer targeting findings using L1 and other chelators, further promotes the prospect of the development of chelators as cancer therapeutics. The mechanistic insights into the interactions of chelators in each cancer type and stage have different characteristics and require different forms of targeting and therapeutic intervention. In particular, effective targeting of metastasis and drug resistance by iron chelators can decrease cancer patient mortality worldwide (Table 2).

## 5. Future Prospects in the Use of Chelating Drugs and Chelator–Iron Complexes in Cancer

**Figure 5:**
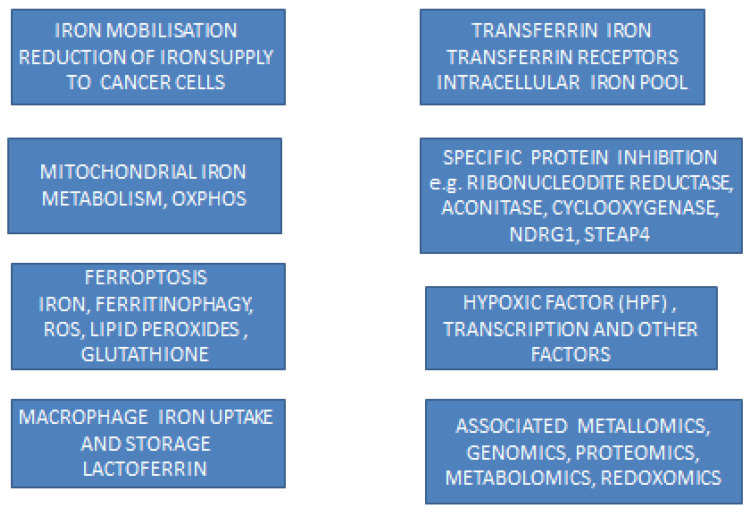
Modulation and targeting by iron chelators of iron metabolic pathways and activities of biomolecules, organelles and cells related to cancer formation, proliferation, metastasis and drug resistance affecting almost all types of cancer. NDRG1: N-MYC downstream-regulated gene-1; OXPHOS: oxidative phosphorylation; ROS: reactive oxygen species; STEAP4: six-transmembrane epithelial antigen of prostate, family member 4.

The identification of new target proteins and transcription, genomic and other molecular factors—as well as new pathways such as ferroptosis, the role of organelles such as mitochondria, the role of cells such as macrophages, the formation and role of free radicals catalyzed by iron and many other new developments related to iron metabolism in cancer—has increased interest in the use of iron and other metal chelators, as well as chelator–metal complexes for targeted treatments in cancer [8,22,60]. In this context, there are encouraging prospects for the use of chelators, including chelating drugs and phytochelators, in many different types of cancer for which current anticancer therapies are ineffective, such as in metastasis and drug resistance, which are the major causes of mortality in cancer patients (Figure 5). In particular, the targeting of iron and copper ions for anticancer activity by chelators such as L1, which can effectively bind both metal ions, is the subject of specific therapeutic strategies for ferroptosis and many different types of cancer [275,276,277,278,279,280,281,282,283,284].

In contrast to iron removal or the inhibition of iron metabolic processes by chelators, the use of lipophilic iron and copper complexes in anticancer strategies is also currently under investigation and development. The introduction of excess iron or copper in some cancer cell types increases susceptibility to cancer cell damage via ferroptosis or other pathways of cellular damage and death. In this context, the approval of the use of the tris-maltol iron complex (ferric maltol or Feraccru or Accrufer) for the treatment of iron deficiency anemia is another major development in the area of chelation and also in relation to anticancer therapeutic strategies, where repurposing specific iron donor drugs can be rapidly introduced in selected types of cancer susceptible to ferroptosis. It should be noted that it has taken almost forty years for iron maltol to reach the clinical stage from the original discovery, which involved initial biochemical and other preclinical studies that proposed the mechanisms of efficient iron delivery from iron complexes to cells, animals and humans [55,285,286,287,288].

Regular monitoring of metabolic changes and tumor markers is essential in assessing the progression of cancer, the selection of treatment and the overall survival of cancer patients. In this context, changes in iron metabolism in cancer patients should be regularly monitored and interventional therapeutic programs including iron chelation could be introduced where appropriate. Increases in serum ferritin, serum iron, transferrin iron saturation, hemosiderin deposition in macrophages and similar changes in cancer patients are negative prognostic features in the progress of the disease. Similarly, excess iron load following repeated transfusions in anemic cancer patients is also a negative prognostic factor and chelation therapy should be introduced [183,184,185,186,289]. Although in these cases, chelation therapy is complementary to anticancer therapy, it may generally improve the therapeutic outcome and long-term survival in cancer patients. 

Therapeutic protocols involving iron chelation and approved anticancer treatments in cancer patients may become a parts of personalized medicine approaches. In the event of chelating drug repurposing as a main or adjuvant therapy in the treatment of cancer, the use of maximum chelating doses may be introduced, similar to the top-range doses used in thalassemia patients (75–100 mg/kg/day L1, 40–60 mg/kg/day deferoxamine, 20–40 mg/kg/day deferasirox) [47,53]. With regard to safety, only L1 is recommended for use in patients with normal or low iron stores, whereas the other chelating drugs are restricted due to toxicity implications [290]. In this context, despite L1 doses of 150 mg/kg/day having been shown to be tolerated in HIV patient studies, the maximum dose of 100 mg/kg/day approved in thalassemia and other iron-loaded patients is initially recommended for anticancer therapy for up to a month in cancer patients [291]. It should be noted that insignificant reduction in the iron stores during a short course, e.g., 2–4 weeks of treatment, is anticipated, similar to many other patient categories with normal iron stores treated with L1, some for as long as 9 months [192,193,194,292,293].

Future drug protocol designs based on the personalized profiles of patients, which take into consideration drug absorption, distribution, metabolism, elimination and toxicity (ADMET) and the metabolomic, genomic, proteomic, redoxomic and other personal biological characteristics of patients, are likely to improve the efficacy and safety of anticancer therapies. Similarly, the selection of new drug formulations and therapies, including iron chelation as a main or adjuvant therapy, is also likely to benefit many categories of cancer patients in the future [17,61,62,63,64,294,295]. In particular, drug combination therapies are increasingly selected instead of monotherapies in many cancer types, which appear to improve the risk/benefit assessment and overall therapeutic outcomes in cancer patients. In such approaches, the characterization of the targets and the determination of the molecular interactions involving iron, and molecular aspects of the therapeutic action of drugs, such as the use of protein–chelator structures determined via X-ray crystallography and other methods, will need full evaluation. Similarly, the identification of more chelator targets, such as the proteins of iron metabolism STEAP1 and lactoferrin, could increase the prospects of anticancer therapeutic activity [224,296,297]. The general use of iron-chelating drugs for controlling iron metabolic pathways in relation to the associated abnormalities, toxicities and diseases can, in many cases, offer improved therapeutic solutions. The therapeutic process, however, is more complex since in such cases, metallomic, redoxomic, genomic, proteomic, metabolomic, pharmacogenomic and other factors can also influence the therapeutic outcome [298,299,300,301] (Figure 5).

## 6. Conclusions

New metabolic mechanisms and pathways related to iron which may affect many diseases, including many types and stages of cancer, have recently been identified. The targeting of these new iron metabolic pathways offers the possibility of new therapeutic interventions in each case, as well as for new universal therapeutic approaches in anticancer strategies using chelators or chelator–metal complex monotherapies, or their combination with established anticancer drugs. In particular, iron-chelating drugs, especially L1, have been shown in many recent studies to fulfill the role of multi-target anticancer drugs linked to new, but also previous targets associated with iron. These targets include the inhibition of DNA synthesis; free radical oxidative damage caused by iron and copper; lipid peroxidation; ferroptosis; modulation of the STEAP4 metalloreductase and metabolic pathways of oxidative damage affecting mitochondria; iron removal of macrophage iron overload affecting anticancer activity; hyperferritinemia; the modulation of hypoxia related to the role of hypoxia-inducible factor (HIF) and HIF PHD; and many others. 

Different therapeutic approaches have been identified for the use of lipophilic chelator–iron or –copper complexes, where the anticancer mechanism is related to chelator’s ability to incorporate iron into cancer cells and initiate toxic free radical cascades including the induction of ferroptosis. This form of targeting is particularly important for some cancer cell types and also in drug resistance and metastatic cancers where, at present, there are no available effective treatments.

The interactions of anticancer drugs with iron and other metal ions such as doxorubicin, bleomycin and hydroxyurea, which are widely used in cancer patients, is also of great importance in relation to their efficacy and toxicity, affecting the morbidity and mortality of thousands of cancer patients. Further studies are needed in this area, as iron-chelating drugs and other therapeutics could play a critical role in improving the efficacy and safety of these and similar anticancer drugs and increase the overall survival of cancer patients. Similarly, effective iron chelation therapies and the elimination of all excess iron in multi-transfused cancer patients, like in thalassemia patients, could also improve the survival of affected transfused cancer patients.

The major role of transferrin in the transport and metabolism of platinum from the use of platinum-based drugs, as well as theranostic and diagnostic drugs containing other metal ions, is another area affecting the majority of cancer patients and where further investigations are needed, including chelation therapy interventions. 

Many targets have been identified in relation to iron and other metal ions where the need for the use of specific chelating drugs or chelator–iron complexes could play a major role in improving the survival and decreasing the morbidity of many cancer patient groups and in the overall majority of cancer patients. In addition, many efforts are still needed in this area, including the identification of optimal doses for maximum efficacy and low toxicity in iron-chelating and other drugs, and also of their combinations.

The low survival rate of cancer patients calls for the need to readdress cancer therapy and consider therapeutic strategies targeting multifactorial processes, including the application of multi-targeting drugs involving iron chelators and iron–chelator complexes. New therapeutic protocols including combinations of established anticancer drugs with L1 and other chelating drugs could increase anticancer activity, improve treatments and reduce toxicity in cancer patients.

## Figures and Tables

**Table 1:** Physicochemical properties of chelators and their iron complexes.

Chelator	Log β	MWt	Kpar	Charge	Kpar	Charge
						Iron complex
Deferiprone (L1)	35	139	0.18	Neutral	0.05	Neutral
Deferoxamine	31	561	0.02	Positive	0.02	Positive
Deferasirox	27	373	6.30	Negative	-	Negative
Omadine	-	127	0.04	Zwitterionic	2.67	Neutral
Tropolone	32	122	3.04	Neutral	4.5	Neutral
8-Hydroxyquinoline	37	145	28.30	Neutral	10.00	Neutral
Mimosine	36	198	0.01	Zwitterionic	0.01	Zwitterionic
Maltol	30	26	1.23	Neutral	0.32	Neutral

Metal stability constants (log β). At physiological pH, all the chelators form a 3 chelator:1 iron complex (log β3) with the exception of deferoxamine, which forms 1 chelator:1 iron complex (log β1) and deferasirox, which forms 2 chelator:1 iron complex (log β2) ([22,26,87,110] and references therein). Molecular weight (MWt ); n-octanol/water partition coefficients (Kpar); charge of chelator and chelator iron complex at physiological pH (Charge).

**Table 2:** Examples of possible uses of chelating drugs and metal complexes in cancer treatment.

**Effects of Metal Ion Mobilization**
Inhibition of cancer cell growth and proliferation through deprivation of essential metal ions from
cancer cells or through metal ion removal from metal-transporting proteins by chelators.
Inhibition of transcription factors through zinc binding by chelators. About 2000 transcription
factors have been identified to be zinc-dependent.
The use of chelating drugs, chelators and metal–chelator complexes as metal theranostics against cancer.
Chelators used for the decorporation of radioactive metals, e.g., uranium, plutonium, and xenobiotic metals such as cadmium and nickel, which cause cancer formation and proliferation.
**Inhibition of Key Proteins Involved in Iron Metabolism**
Inhibition of the iron-containing enzyme ribonucleotide reductase, which is involved in the reduction
of ribonucleotides to deoxyribonucleotides in DNA synthesis.
Inhibition of transferrin receptor uptake of iron transferrin, which is up-regulated in many cancer types, e.g., breast cancer, prostate cancer and leukemia.
Inhibition of aconitase activity, which affects mitochondrial metabolism and function and is also crucial for tumor proliferation, survival and metastasis.
Inhibition of free radicals and other regulatory molecules produced during cycooxygenase and lipoxygenase activity.
Modulation by chelating drugs and other chelators of the metastasis suppressor N-MYC downstream-regulated gene-1 (NDRG1), six-transmembrane epithelial antigen of prostate, family member 4” (STEAP4) protein, hypoxia-inducible factor (HIF) and similar biomolecules.
**Inhibition of Free Radicals and Antioxidant Action**
Inhibition of free radical cascade toxicity formed by iron and copper catalytic centers, causing damage and modification to DNA and other biomolecules.
Chelating drugs for preventing free radical toxicity including tissue damage following radiotherapy and chemotherapy.
Design of drugs and protocols, e.g. dexrazoxane and L1, for protection against cardiotoxicity of the anticancer drug doxorubicin.
**Modulation of Ferroptosis and Associated Processes**
Inhibition of ferroptosis by non-redox-active chelating drugs such as deferiprone and deferoxamine and other chelators with similar properties.
Induction of ferroptosis by redox-active lipophilic chelators and iron complexes
Induction of ferroptosis by redox-active lipophilic chelators and iron complexes in combination with reducing agents such as ascorbic acid.
Ferritin iron mobilization during ferritinophagy in ferroptosis.
Increased macrophage anticancer activity via iron mobilization from hemosiderin and ferritin in iron-laden macrophages.
**Modulation of Targeting Activity**
Design of chelators for cell cycle control and prevention of drug resistance and metastasis.
Design of new anticancer drugs of equivalent or greater efficacy in cytotoxic activity in comparison to existing drugs, e.g., omadine and derivatives.
Combination protocols of established anticancer drugs with chelating drugs. Synergistic effects and better efficacy overall in anticancer activity are observed in comparison to monotherapies.
Design of inactive prodrug that can be converted to active chelating drug for targeting specific active pathways in cancer cells but not normal cells.
Design of chelating drugs with different partition coefficients for the targeting of either lipophilic or hydrophilic compartments in cancers cells.
**Therapeutic Effects Improving the Survival of Cancer Patients**
Chelating drugs, e.g., deferiprone, deferoxamine and deferasirox, for preventing iron overload toxicity in regularly red-blood-cell-transfused cancer patients.
Chelating drugs for preventing infections in immunocompromised patients following chemotherapy and radiotherapy.
Chelator–iron complexes used as monotherapy, or in combination with erythropoietin, for the treatment of chronic anemia in cancer patients.

## Abbreviations

ADMET	absorption, distribution, metabolism, elimination and toxicity
L1	deferiprone
HIF	hypoxia-inducible factor
HIF PHD	hypoxia-inducible factor prolyl hydroxylases
MRI	magnetic resonance imaging
NDRG1	N-MYC downstream-regulated gene-1
OXPHOS	oxidative phosphorylation
PET	positron emission tomography
STEAP4	six-transmembrane epithelial antigen of prostate, family member 4
